# Designing a Participatory Total Worker Health^®^ Organizational Intervention for Commercial Construction Subcontractors to Improve Worker Safety, Health, and Well-Being: The “ARM for Subs” Trial

**DOI:** 10.3390/ijerph17145093

**Published:** 2020-07-15

**Authors:** Susan E. Peters, Hao D. Trieu, Justin Manjourides, Jeffrey N. Katz, Jack T. Dennerlein

**Affiliations:** 1 Harvard Center for Work, Health, and Well-Being, Harvard T.H. Chan School of Public Health, Boston, MA 02115, USA; 2 Dana-Farber Cancer Institute, Boston, MA 02115, USA; 3 Bouvé College of Health Sciences, Northeastern University, Boston, MA 02115, USA; h.trieu@northeastern.edu (H.D.T.); j.manjourides@northeastern.edu (J.M.); 4 Departments of Orthopedic Surgery and Medicine, Brigham and Women’s Hospital, Boston, MA 02115, USA; jnkatz@bwh.harvard.edu; 5 Department of Epidemiology, Harvard T.H. Chan School of Public Health, Boston, MA 02115, USA

**Keywords:** construction industry, occupational health, workplace, health promotion, ergonomics, musculoskeletal pain

## Abstract

*Background:* Evidence supports organizational interventions as being effective for improving worker safety, health and well-being; however, there is a paucity of evidence-based interventions for subcontracting companies in commercial construction. *Methods:* A theory-driven approach supplemented by formative research through key stakeholder interviews and focus groups and an iterative vetting process with stakeholders, resulted in the development of an intervention for subcontractors in the commercial construction industry. We piloted the intervention in one subcontracting commercial construction company. We used these findings to adapt and finalize the intervention design to be tested in a future large-scale trial. *Results:* There were several key findings from the formative research, including challenges faced by companies and assets that should be considered in the intervention design. This resulted in a communication infrastructure company-based, continual improvement, participatory intervention design, consisting of a needs assessment and report, committee-led prioritization, action planning and implementation, and worker communication/feedback cycle. The pilot contributed to the final intervention design with modifications made with respect to timing, implementation support, capacity building, adaptability and sustainability. *Conclusions:* The use of a theory-driven participatory approach to developing an integrated organizational intervention for commercial construction subcontracting companies was important and necessary. It allowed us to consider the empirical evidence and relevant theories and tailor these to meet the needs of our target population. This study gives pragmatic insight into the early development of a complex intervention, with practical experience of how we adapted our intervention at each stage. This intervention will be tested in a future randomized trial.

## 1. Introduction

There is a large need to address the high rates of chronic health issues of construction workers through Total Worker Health^®^ (TWH^®^) approaches [1], especially those that target the conditions of work [2]. Construction workers suffer high rates of musculoskeletal disorders [3,4,5,6], obesity [7], smoking, alcohol consumption and substance use [8,9,10,11], as well as poor mental health and high risk of suicide [12,13,14]. These poor health outcomes are influenced by the conditions of work, including work organization (such as break-taking practices, seasonal work, production demands), hazardous work environments and high physical and mental job demands [15,16,17]. In the construction industry, there has been a large emphasis on workplace injury prevention, but little effort addressing other workplace health risk factors through an integrative approach involving ecologic systems-level upstream approaches targeting the conditions of work [2,18]. 

Implementing systems-level integrated approaches in the construction industry has many challenges related to the industry’s structure and employment practices. Like many modern fissured workplaces, a construction site contains multiple employers [19,20,21]. For a given construction project, the project owner hires a general contractor, who is responsible for coordinating work and worksite safety. They, in turn, hire subcontractors to perform about 90% of the site’s trade-specific work; thus, the subcontractors are the main employers on site [22]. These subcontractors are obligated to provide employees with the resources to complete the job safely and efficiently, as well as providing health insurance benefits. When a worker enters a worksite they usually do so as part of a team of workers from a given subcontractor; however, once on site they are embedded in this multi-employer structure, working alongside other workers from other subcontractors all under the auspices of the general contractor [23,24]. 

Adding to this worksite complexity, workers within a subcontracting company can be distributed across multiple worksites, each with their own safety and health culture. Thus, it can be difficult to reach workers consistently and in a timely manner. Communications between workers and managers related to worker safety and well-being, as well as work quality and productivity, can be limited [25]. This challenge is exacerbated by many subcontractors being small employers having limited resources and manpower devoted to worker health and safety. In a national database of U.S. subcontractors, 75% have fewer than 200 employers [26]. 

In our previous worksite-based program for general contractors, “All the Right Moves (ARM)” we found that an intervention focused on both health promotion (through toolbox talks) and health protection (through worksite ergonomic evaluations and changes) was effective in improving worker safety and health behaviors [27]. However, our data suggested that the ARM program could have been more effective if the program included resources and activities specifically targeting the subcontractor. Workplace approaches may not have the desired effect, as workers are onsite for relatively short periods [28]. 

The aims of this paper are to: (i) present a TWH^®^ intervention design for subcontractor companies in the construction industry that addresses many of the lessons learned from a previous worksite program that we developed for general contractors; (ii) outline the design process that led to this intervention; and (iii) describe the logistic considerations of implementing the intervention with a pilot company.

## 2. Materials and Methods 

Our multi-disciplinary intervention development team, as well as an external advisory board consisting of experts from construction research, organizational intervention design, ergonomics, public health, allied health and medicine, developed this intervention. We met and corresponded regularly during a sixteen-month intervention development in 2016–2018. The human subjects research protocols and consent procedures were approved by the Harvard T.H. Chan School of Public Health Institutional Review Board (IRB13-1948; CR13-1948-08). 

### 2.1. Theory-Based Models Used to Guide Intervention Design

We used principles from community-based participatory research methods [29], and intervention mapping [30], to guide the development process. The intervention process itself was based on both the Harvard Center for Work Health and Well-being conceptual model (‘Harvard Center’s conceptual model’) [2], and the key steps for integrated approaches for organizational health and safety interventions outlined in the Harvard Center for Work, Health, and Well-being’s Guidelines [18]. These guidelines provided a step-by-step guide to develop integrated health and safety organizational interventions based on the theoretical framework of TWH^®^ approaches as well as practical considerations [18]. The Harvard Center’s integrated approach to workers’ safety, health and well-being is a strategic and operational coordination of policies, programs, and practices designed to simultaneously prevent work-related injuries and illnesses and enhance overall workforce health and well-being. 

### 2.2. Formative Research Conducted to Inform Intervention Design

We conducted formative research to determine what characteristics needed to be considered for the success of the intervention and its implementation, and to establish the intervention components and their methods. The focus of the formative research was on the adaptation of a previous and successful worksite-based intervention [27], and determining the feasibility and practical application of the intervention components. 

We completed key informant interviews with general managers and safety managers from subcontractors from various trades in Massachusetts to learn more about subcontractors’ employment patterns and to determine effective strategies for implementing and evaluating a TWH^®^ organizational intervention to improve subcontracting workers’ safety, health and well-being. Participants were recruited through our existing construction projects from a previous study [27]. Semi-structured interviews were conducted in-person and were recorded, transcribed and content analyzed using Atlas.ti 7 Windows (Atlas.ti Scientific Software Development GmbH, Berlin, Germany). 

### 2.3. Designing the Intervention 

We drafted an intervention based on our formative research. The intervention was continuously reviewed and refined through an iterative process based on feedback from construction and Center stakeholders. We revised the protocol several times until consensus across Center stakeholders was achieved.

#### Vetting and Refining the Intervention Design with Key Stakeholders and Construction Workers 

We vetted the intervention components, activities, timing, delivery and implementation methods with union representatives, and subcontractor safety and human resource representatives through interviews, as well as two focus groups—one with general contractor and subcontractor representatives and another with managers and workers from one subcontracting company. Participants were recruited from our previous and existing construction projects [27,31]. To test out new ideas during this vetting process, we made changes to the intervention protocol sequentially, and hence no two touch points reviewed the same intervention protocol. All interviews and focus groups were conducted in-person and were guided by a semi-structured interview guide. The sessions were audio-recorded and transcribed. We then analyzed the content of the responses to the interview/focus groups questions (i.e., intervention content, activities, timing, delivery and implementation methods, other overall comments) using NVivo 11 Mac (QSR International, Melbourne, Victoria, Australia).

### 2.4. Piloting the Intervention

We pre-tested the draft intervention protocol with a Massachusetts commercial construction subcontracting company during 2018. The goal of the pilot was to ensure that the intervention activities and processes aligned with our target population and contextual factors that may influence feasibility and adoption prior to larger scale testing. We instructed the company to follow the intervention protocol as it was designed; however, they were also able to provide input at every step with respect to what was working well and what could be improved. We documented all process data, including any deviations from the protocol and reasons why, using an activity log. 

We also collected data from key informant interviews using a semi-structured guide at two timepoints during the pilot: at the midpoint after completing the continuous improvement cycle of the draft intervention once, and then again after the draft intervention cycle was completed a second time. The key informants included three foremen, two project managers, one apprentice, and one general site superintendent from the subcontracting company. The interviews explored the perceived benefits of the program; program feasibility, acceptance and sustainable intervention program components; what components worked well and what components did not work well; suggestions for improvements; and contextual factors that might need to be considered in the final intervention design.

## 3. Results

### 3.1. Key Findings from our Formative Research

Our key findings fit into two categories: (i) challenges faced by subcontractors, and (ii) assets that could contribute to successful intervention implementation (Table 1). In synthesizing these data, we found that many of the activities of the original ARM worksite-based intervention would not work for the subcontracting companies. Thus, the design was changed to a company-based intervention to accommodate the unique characteristics of subcontracting construction companies. 

### 3.2. Drafting the ARM for Subs Intervention 

**Figure 1:**
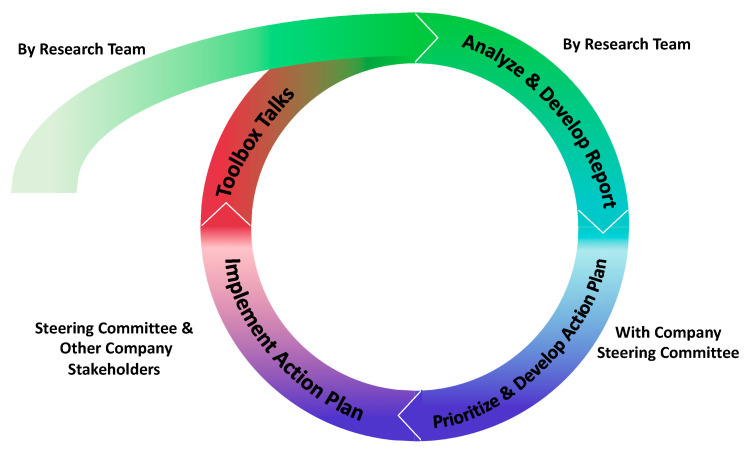
Continuous improvement intervention cycle.

We drafted a protocol for our ARM for Subs Intervention that would then be vetted with a pilot company. This protocol included a manual and accompanying materials to guide and conduct the activities, as well as provide training and resources to the company. The initial intervention design consisted of five main sequential activities structured within a continuous improvement cycle through a communication infrastructure between front line workers and managers (Figure 1). The goal of each cycle is to remediate a prioritized problematic working condition, which had been identified by workers, by implementing changes to company policies, programs and practices, and then communicating these changes back to workers. The research project program has two continuous improvements cycles—the research team would facilitate the first cycle, and the company would facilitate the second cycle with the research team providing technical assistance. The company then should sustain the program by continuing the cycles without assistance from the research team. We anticipated that each cycle would be approximately 6–8 weeks in length. 

#### 3.2.1. The Continuous Improvement Cycle with the Specific Intervention Components 

**Figure 2:**
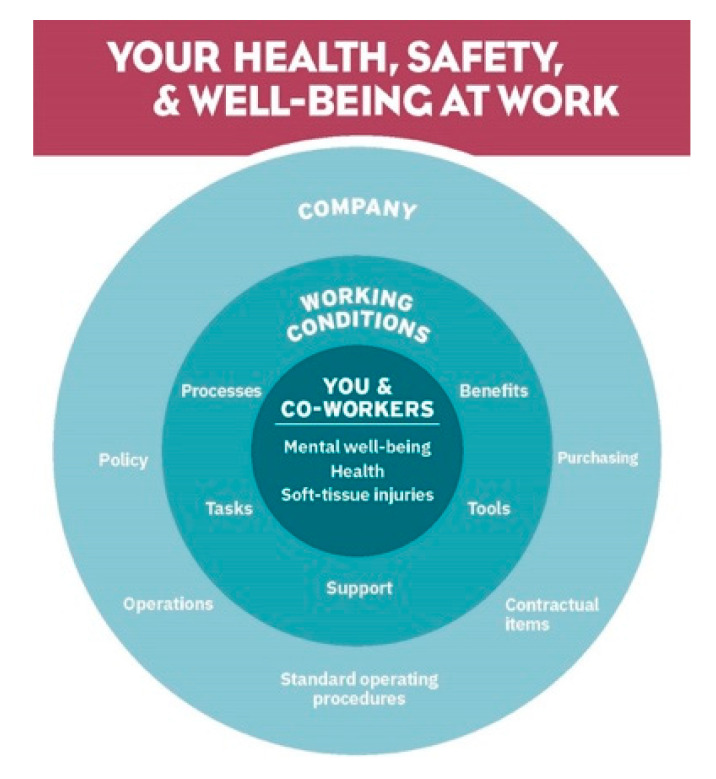
The socio-ecological framework to guide toolbox talk discussion and organize topics for the report.

The needs assessment should utilize a series of three discussion-based interactive toolbox talks (ITTs) to collect qualitative data to determine the safety and health experiences of the front line workers. Each talk is facilitated by a member of the research team, designed to take fewer than 15-minutes and conducted over three consecutive days. The facilitator uses an open-ended guide with a supplementary poster (Figure 2) to facilitate worker involvement based on a social ecological theory model [32].

The first ITT would provide an overview of the Program. The second would focus on collecting information about worker safety, health, and well-being issues. Workers are asked about specific working conditions or injuries that either are caused or exacerbated by work. The third would focus on collecting information on working conditions and company polices, programs, and practices that impact their health, safety and well-being. 

During the ITTs, workers are encouraged to write down both “areas for improvement” and “things they are proud of” using index cards and place these in a locked boxed accessed only by the researchers anytime during the three days. Including positive factors provides opportunities to identify potential solutions [33]. In addition, notetakers could record the ideas that workers verbalize during the ITTs. The locked box and blank index cards should be kept on-site for three days, allowing workers to provide feedback outside the ITTs. This should create an environment in which workers would feel comfortable providing earnest feedback. At a later time, the research team then creates a report summarizing the data collected. Through an iterative thematic analysis process, we would categorize utterances using the socio-ecological framework (Figure 2) until consensus is reached among the research team. As workers were writing on index cards, a consensus approach was taken to ensure accurate interpretation of the workers’ responses. 

At the same time as the needs assessment report is being developed, we would work with the company to create a steering committee. The committee should prioritize and create action plans to address topics and coordinate and communicate with key company personnel to ensure leadership commitment is maintained. The steering committee should have fewer than 12 members and include workers and managers from relevant divisions of the company. 

The researchers would facilitate the first meeting with the steering committee, where the committee receives in-person training consisting of an overview of the intervention including the goals, process, key personnel roles and responsibilities and key activities; the prioritization and action planning process to change working conditions; and the importance of worker communication process and participation. Then, the committee would review the report and prioritize one key working condition to target each cycle. In the second meeting, the committee should identify one area that they will undertake first using an infographic resource to guide which conditions will be both impactful to workers and the company, and feasible to implement in a timely way (i.e., looking for a “quick win”). During this process, employees and managers work together.

At the following meetings, the committee should create action plans by identifying and implementing strategies addressing the prioritized topic areas, with a focus on improving problematic working conditions through changes in policies, programs and practices. Meeting every 2–4 weeks, the committee should continue to brainstorm strategies and develop specific, measurable, actionable, feasible and timely action plans outlining clear objectives, key implementation steps, personnel responsible, and timelines to successful implement strategies.

The committee should establish a mechanism for communicating with key stakeholders as well as communicating with workers about proposed changes. The goal is to provide frequent communications with both leadership and workers to ensure participation and buy in from both groups to increase the likelihood of successful implementation of the action plan. This would ensure that senior leadership is informed, allowing them to provide feedback and dedicate resources and to make the required changes. To communicate to workers, one to three TTs on the priority topic areas should be conducted. A set of talks already existed from our previous interventions, and are available to be utilized for this purpose [27]. In addition, we planned to develop new TTs or use existing ones which are available publicly. 

#### 3.2.2. Cycle 2 and Sustainability of the Program

To build the capacity of the company to sustain the program, the company leads the second cycle on their own, with only technical assistance from the research team. The company should use either the needs assessment report to select topics not chosen during the first cycle, or it could conduct a new needs assessment. 

### 3.3. The Pilot

#### 3.3.1. Company and Worker Sample

The pilot involved one small, family-owned and operated sub-contracting excavation company with 20 construction workers across two worksites in Massachusetts, USA. This is representative of the majority (80%) of U.S. companies in construction, being a small business [34]. A further 60% of USA construction workers are employed in companies that have fewer than 50 employees [35]. The company has worked with a variety of general contractors, from large unionized companies to small independent firms. Workers were unionized and worked across three trades—carpenters, operators and laborers. Employees had completed at least high-school-diploma-equivalent, trade-specific training, as well as OSHA-30 training. Depending on the work, the company retains a small group of workers and then hires new workers, often from local union halls, depending on the size of contracts. A total of 20% of workers who participated in the needs assessment were female. However, the steering committee was all male, except for one. Most workers onsite spoke English (and all pilot activities were conducted in English). However, we noted that several workers spoke Spanish and their coworkers translated for them during the Pilot.

#### 3.3.2. Pilot Cycle 1

The needs assessments took 2 weeks to coordinate and complete, as workers were distributed across two sites, thus a total of six talks was required (Site 1: ITT 1 (*n* = 15), ITT 2 (*n* = 8), ITT 3 (*n* = 16); Site 2 ITT(*n* = 8), ITT 2(*n* = 8), ITT 3 (*n* = 8)). Due to the constraints of the company, two of the ITTs were conducted on the same day. The needs assessment resulted in 93 responses of issues grouped into several topic areas, such as toxic effects caused by exposure to unknown chemicals in the air and ground, lack of communication for resolving safety concerns raised by workers contributing to workers’ stress, lack of workplace smoking policies, stigma related to accommodating health/injury at work, and health concerns related to meal break policies and lack of dedicated areas. 

After the report was generated, company management requested a review of the report prior to the first steering committee meeting. This helped to establish credibility with the company management and buy-in for the program moving forward.

The company developed a committee specifically for the program, consisting of five managers and four workers. The first meeting was at one of the two job sites. After the first steering committee meeting, the company appointed one of the foremen and a project manager to co-lead the committee. At the first meeting, the committee was provided brief training as per the protocol. The committee discussed developing communication mechanisms to key decision makers at the management level, and to workers, to provide information on what changes were being made, establish what resources were available for solutions and to gather further information and feedback about the targeted area. The committee decided to communicate the proposed changes to workers via toolbox talks (TTs) at the completion of each cycle. The committee prioritized three main health concerns and their related working conditions—two to be addressed in Cycle 1 and one in Cycle 2. 

Action Planning commenced at a second meeting held three months after the first meeting at a different jobsite from the first meeting. There were two managers and five workers in attendance, two of which were new to the steering committee. We provided the committee with training on action planning and an action plan template to guide the process. We worked with the steering committee to establish mechanisms to engage the appropriate people in the planning process. These included key decision makers from within the company and with the sites’ general contractor for the specific action plans, and those who needed to approve resource allocation for the plans as well as a timeline for the action items on the plans. The action-planning phase in Cycle 1 took approximately one month.

Two topic areas were addressed within Cycle 1, as one concern could be addressed by a simple, low-cost solution almost immediately, and another was considered the most important topic that also needed to be addressed in a timely manner:

1. Health effects caused by lack of clean and dedicated areas for meal breaks: This was selected as a short-term “quick win” for the Program that could be easily remedied with a low-cost solution. The plan was quickly agreed upon and implemented shortly thereafter. The operations manager was responsible for purchasing a portable table and chairs to create a clean meal area that could be moved from site-to-site. This was implemented within a week;
2. Toxic effects caused by exposure to unknown chemicals in the air and ground during excavation: This was selected as the most important topic area by both workers and managers due to its long-term health consequences and the stress that it was causing workers. Potential solutions were more complex and required changes across multiple systems and organizational levels at the worksite, such as engaging the site’s general contractor. During the planning, the committee realized that the contract between a subcontractor and the general contractor contains information regarding policies for air and soil sampling, and how and what is communicated by the general contractor to the subcontractor. Providing detailed information raised liability considerations from their insurance company—i.e., what are the implications if a certain contaminant is not known at the time and hence not communicated to workers? As a result, two alternate solutions were pursued. One “quick-win” to reduce the uncertainty of exposure was to provide facilities for workers to change out contaminated clothes before leaving the site. Again, the operations manager was responsible for implementing this solution. Another longer-term solution was to update the company’s policy in their safety handbook to allow workers to stop work when a worker identifies a potential hazard from the ground or in the air and to notify the operations manager to escalate and resolve the issue. The specific updated page from the handbook was then printed and placed in employees’ pay-checks so that all workers were told the new policy.

Two TTs related to these solutions were provided to 20 workers across two jobs sites over two days at the end of Cycle 1. The TT communicated the topic areas being addressed to workers and obtained worker input on the potential solution. In addition, the steering committee was provided with our series of topic-based TTs for use on-site. 

#### 3.3.3. Pilot Cycle 2

The company changed the location of the steering committee meetingsfrom on-site to their field office at the same time as the management meetings and gave primary responsibility to the operations manager to lead the committee and its meetings. The research team provided support to the operations manager, including reviewing and providing input on action plans, evaluating the success of action plans and responding to queries about worker participation. 

For the second cycle, the committee addressed the third topic, which was lack of communication for resolving safety concerns raised by workers contributing to workers’ stress. The workers identified that they were stressed and not as productive due to lack of communication and follow-up on raised safety issues, unaddressed reports of safety concerns, disorganized site plans resulting in safety concerns (e.g., where the digging locations were mapped out; where holes were initially covered but not identified as a fall hazard and someone fell). These various health and safety concerns were addressed by: (1) implementing a red tag/green tag system for functioning /not functioning equipment; and, (2) a new Safety Opportunity Program—a less aggressive and no finger-pointing near-miss reporting form to share safety concerns with the company. The company rolled out the program by including the Safety Opportunity Forms in their employees’ paychecks. Workers were encouraged to complete the form and drop it off in a suggestion box that the company purchased, or it could be passed directly to the lead foreman. At the end of each week, the site superintendent collected the completed forms and the project manager entered the information into a spreadsheet to track if the issues were addressed or not via their weekly safety meetings at the field office. The goal of this program was to encourage and promote low-stress communication so that workers could be more productive in the field and know that managers are holding themselves responsible by keeping a tracked log of all communications. This cycle took about three months.

#### 3.3.4. Sustainability of the Program at the Pilot Company

The research team completed quarterly check-in phone calls with the company in the two years following the pilot. The pilot company has continued to adapt the program to fit their needs and build on processes that are working well and receiving positive feedback from workers. Since the pilot program they have included “Health and Safety Moments” (about 10 to 15-min each morning) incorporated into weekly meetings both in the office and the field. Employees have the opportunity to identify health and safety concerns for the day and are encouraged to share the reasons that they choose to do to be healthy and work safely. The pilot company’ steering committee has also created a “why safety matters” bulletin board which is updated regularly. 

The company also adapted the needs assessment form and conducts a similar activity with workers twice a year during company outings (July-August and December). The company reported that they felt that the company outings provided a “safe space” for workers to provide feedback and suggestions for improvements. The pilot company has continued the steering committee meetings on a quarterly basis—not as frequent as the program’s timeline of twice a month. 

### 3.4. Lessons Learned from the Pilot 

Various changes were made during the pilot and to our final intervention protocol (Table 2).

1. Timing: Our anticipated timeline needed to be extended to 12 weeks as each cycle took approximately three months, instead of the intended 6–8 weeks. This was mainly due to the logistics of setting up meetings and toolbox talks as well the iterative process necessary for action planning for more complex topics. This was also complicated by distributed crew locations, inclement weather, and project timing. Of the three priority topics, one was a quick win, while the other two were more complicated and required more time to go through the iterative steps of brainstorming and determining the feasibility of potential solutions;
2. Implementation support: We learned that balancing the amount of information we gave the steering committee at any time was important—too much upfront and they felt overwhelmed, but not enough and they felt lost in the process. A delivery method and format for resources (e.g., in-person training as well as written materials) were needed. Thus, we created FAQs and tip-sheets in simple language for how to complete each of the steps and key intervention activities. Supporting materials needed to be straightforward, easy to follow and brief due to the dynamic and fast nature of construction.
3. Tailoring to fit the company: A benefit of the intervention was that it was able to be flexible to the companies’ needs and was able to be adapted to fit within the current systems. All companies have their own characteristics and systems in place. An intervention such as this one should consider using an existing committee to serve as the steering committee to streamline meeting times and locations; and identify other mechanisms for conducting needs assessments that could be easily implemented by companies themselves, e.g., worker quarterly surveys distributed with their pay-checks. We also realized that companies may need to modify our resources to according to their own needs and preferences;
4. Capacity building: The company needed a minimum level of training and technical assistance to be able to complete the ARM for Subs program. Companies have limited time during the day to participate in the intervention activities in addition to their usual workload. If you provide too much training, it takes too much time and they also feel overwhelmed; too little, and they do not feel well informed and potentially lose trust in the process. During key informant interviews, steering committee members stated that they benefitted from a research-led cycle followed by a researcher-coached cycle, prior to taking over the ARM for the Subs program themselves.
5. The universality of building communication processes within the company: One of the benefits reported was that the company “learned more about their own company”—both the areas that were working well, and areas that could be improved. They also learned that communication was integral to ensuring leadership commitment to the program and worker buy-in. TTs were moved to earlier in the intervention cycle so that they were delivered in the week after the report was generated, rather than at the end of the intervention cycle. The company reported that workers provided them with constructive information that could benefit the company by enhancing workers safety and health systems, and also quality and productivity outcomes.
6. Adaptability and sustainability: Exploring structures that already existed and could be tapped into by the steering committee, allowed the company to see that the approach used in this program could be easily integrated and potentially sustained. The cyclical design of the intervention allowed us to demonstrate to the company the processes in the first cycle, with the second cycle allowing the company to adapt and ‘own’ the process, which helped to demonstrate the sustainability of the program over the long-term.

## 4. Discussion

The goal of this paper was to provide a real-world example of how an intervention was adapted from research and implemented in practice by describing the key steps taken to develop an employer-based intervention to improve worker safety, health, and well-being for subcontracting companies in the commercial construction industry. The process and systematic development of a complex multifaceted intervention provided a path to implement a participatory TWH^®^ intervention approach in small businesses within the fissured workplace structures of the construction subcontracting industry.

Our intervention development approach drew on several evidence-based frameworks and studies in the construction industry and used a modified intervention mapping approach to “fit” both the theory and evidence into a pragmatic construction setting. This resulted in an intervention design which consisted of a participatory needs assessment, prioritization and objective setting, action planning, and worker training steps as part of a continuous improvement model that was adapted to meet the unique contextual factors of subcontracting companies. These factors included distributed and itinerant workforces working in multi-employer workplaces, under-developed safety management systems and the limited human and financial resources often inherent in operating small businesses. 

We were constantly re-evaluating and learning how our intervention worked and how it needed to be adapted. By systematically incorporating empirical findings, theories, and input from stakeholders, the intervention design changed over time and considered the contextual factors of the fissured nature of the construction industry and the diverse set of tasks, hazards, and working conditions. The approach in developing this intervention can be applied to other industries with similar contextual characteristics of fissured workplaces. The core intervention components were based around a communication infrastructure which was developed in such a way that they could be adapted to either be integrated within existing management systems and processes or developed as a standalone safety management system if this did not already exist, regardless of the industry/trade. This finding, that context matters, has been identified across other industries [36]. For example, in a TWH^®^ organizational intervention in the food service industry, several cafeterias owned by the same company but situated within different client organizations were found to have different safety and health issues, even though they provided the same service [37].

Participatory and communication methods were a key part of our intervention design approach and led to new insights that would not otherwise have been discovered [30]. Participatory methods are critical in developing and implementing both public health and occupational health interventions [38,39]. Our intervention considers workers as key information and change agents by including them as members of the steering committee, and through a communication feedback loop in which information is derived from and delivered back to the workers. Workers are often a company’s greatest asset; however, they are generally underutilized by organizations as a resource for continual improvement, especially with respect to their own workers’ health and safety. The specific conditions of work to be addressed by the committee were informed directly by workers. This engaged workers in the intervention itself, which further enhanced the pilot’s success and ultimately worked towards ensuring worker buy-in. Participation from management was also key to ensuring leadership commitment through the action of providing resources to improve working conditions through new and updated policies, programs and practices [40]. Participatory action planning allows workers and managers to develop a shared mental model of the problem and solution [41].

Adaptability and flexibility in our approach was critical in fitting the intervention to the company. The intervention is a communication infrastructure that facilitates a discovery and planning process where the workers and managers identify topics and create plans to address these topics. The specific activities that make up the communication infrastructure can be moved and adapted without compromise to the location of workers and the structure of the company. 

Our formative research allowed us to explore the limitations that small- to medium- sized companies face, as well as assets that could be incorporated and built upon in the intervention design. This is a significant gap in the literature as small- to medium-sized companies have higher workplace fatality and injury rates than larger companies [42]. Many barriers for smaller companies implementing occupational safety and health initiatives exist: stringent legal requirements and administrative workload associated with these initiatives; lack of technical support; involvement of trade unions; lack of standardized guidelines; lack of management commitment and worker buy-in; management and personnel feeling like they are not adequately skilled to implement such initiatives; lack of time and economic resources; absent or ineffective communication; prioritization of productivity over safety; and, absent or ineffective methods for collecting data [43,44,45]. Our company-based approach enabled a small company to develop a safety management system that could be integrated into their day-to-day functioning. It has been recommended that the most successful intervention models for small- to medium-sized companies need to be tailored, action-oriented, low-cost approaches, combining health and safety with other management goals, and based on trust, participation and consistent communication [46,47].

A limitation was that we piloted the intervention in only one company. As subcontracting companies can vary considerably across different trades, piloting across different companies and trades may have resulted in further adaptions to our intervention protocol. However, we developed our intervention to be flexible in response to the perceived needs of future companies. In addition, we did not evaluate the intervention with work surveys and specific outcome metrics. The intervention described here will be tested in a future trial and will be reported elsewhere. 

## 5. Conclusions

The use of a theory-driven participatory approach was a suitable model to achieve the important and necessary goal of developing an integrated organizational TWH^®^ intervention to improve worker safety, health and well-being for commercial construction subcontracting companies. It allowed us to consider the empirical evidence as well as relevant theories and tailor these to meet the needs of construction workers employed through subcontractors. The pragmatic insight with the practical experience of how we adapted our intervention at each stage allowed us to fit the theory with the practice of these organizations (i.e., intervention-organization fit) addressing key implementation details to effectively convey the intervention process of increasing worker voice in a very top-down heavy industry with significant issues related to worker safety, health and well-being. 

## Figures and Tables

**Table 1:** Main findings from our worksite-based research and key informant interviews of subcontracting companies and other context experts.

Challenges Faced by Subcontractors	Assets That Could Contribute to Successful Intervention Implementation
- Large proportion are small employers often without HR and safety professionals - Limited resources to devote to safety and health initiatives (time, people, money) - Productivity prioritized over health and well-being to keep jobs on track - Distributed workforce across multi-employer worksites - Workers move with the work: bid-to-bid, site-to-site - Safety, health and well-being concerns vary between trades and, within the same trade, no two companies are alike	- Direct employers of the workers—feelings of accountability and responsibility to workers - Fewer hierarchical levels when developing an intervention within the company structure (rather than based at a worksite) - Worker solidarity within the trades and teams that move across sites together - Asking for help to improve worker health and safety is more accepted at the company level—*“Health and safety is everyone’s business.”* - Workers are a company’s greatest asset—they have the most knowledge on which working conditions affect safety, health and well-being the most while out on site

**Table 2:** Description of the intervention components and refinements made informed by the pilot.

Intervention Components	Description of Activities	Anticipated Timing	Adapatation Made during Pilot	Future Recommendations Based on Pilot
Needs Assessment (NA) to determine workers’ experiences	ITTs over 3 days facilitated by research team	Week 1	ITTs were conducted over 2 weeks due to multiple sites. Some sites had 2 ITTs on same day	Allowed flexibility in ITTs timing to meet company’s needs
				Provided resources for company to complete their own NA
Develop Steering Committee (SC)	Workers and managers invited to participate in an intervention SC	Week 1	SC composition and location changed to work with existing structures	Adapted SC so company can use existing committee if one is present
Report generated by Research Team	Using NA data, a report is generated using a systematic iterative process	Week 2	No adaptations were made to the report. However, an we added a meeting to review the report with company leadership prior to the first SC meeting.	Add review of the report by company leadership prior to first SC meeting
Prioritization of intervention topics by SC	Training on prioritization and action planning process	Week 3	Company selected more than one topic area per cycle as some topics were quick wins and some required longer.	Company can select more than one topic area per cycle.
	Review report			Developed FAQs and tip sheets to support in-person training
	Prioritize intervention focus area			
Action planning and implementation of strategies	Steering committee meetings to develop action plans	Weeks 4–8	Action planning took approx. three months to complete a cycle	Increased time for each cycle
	Implement action plans between meetings			
Toolbox Talks (TT) with workers	Toolbox talks on NA topics	Week 8	No adaptations made	Moved TTs earlier in the intervention cycle
	Toolbox talk to communicate action plans		Developed new TTs based on company’s priority NA topics	Developed new TTs based on company’s NA topics
Sustainability of the program	Continuing the cycles	After the program	Company adapted activities to fit with existing systems and structures	Developed FAQs/tipsheets for companies on how to complete activities themselves
